# Understanding water conservation vs. profligation traits in vegetable legumes through a physio-transcriptomic-functional approach

**DOI:** 10.1093/hr/uhac287

**Published:** 2022-12-29

**Authors:** Pingping Fang, Ting Sun, Arun Kumar Pandey, Libo Jiang, Xinyang Wu, Yannan Hu, Shiping Cheng, Mingxuan Li, Pei Xu

**Affiliations:** College of Life Sciences, China Jiliang University, Xueyuan Street No.258, Hangzhou 310018, China; College of Life Sciences, China Jiliang University, Xueyuan Street No.258, Hangzhou 310018, China; College of Life Sciences, China Jiliang University, Xueyuan Street No.258, Hangzhou 310018, China; School of Life Sciences and Medicine, Shandong University of Technology, Xincun West Road No.255, Zibo 255000, China; College of Life Sciences, China Jiliang University, Xueyuan Street No.258, Hangzhou 310018, China; College of Life Sciences, China Jiliang University, Xueyuan Street No.258, Hangzhou 310018, China; Henan Provincial Key Lab of Germplasm Innovation and Utilization of Eco-economic Woody Plant, Pingdingshan University, Weilai Street No.1, Pingdingshan 467000, China; College of Life Sciences, China Jiliang University, Xueyuan Street No.258, Hangzhou 310018, China; College of Life Sciences, China Jiliang University, Xueyuan Street No.258, Hangzhou 310018, China; Key Laboratory of Specialty Agri-Product Quality and Hazard Controlling Technology of Zhejiang, China Jiliang University, Xueyuan Street No.258, Hangzhou 310018, China

## Abstract

Vegetable soybean and cowpea are related warm-season legumes showing contrasting leaf water use behaviors under similar root drought stresses, whose mechanisms are not well understood. Here we conducted an integrative phenomic-transcriptomic study on the two crops grown in a feedback irrigation system that enabled precise control of soil water contents. Continuous transpiration rate monitoring demonstrated that cowpea used water more conservatively under earlier soil drought stages, but tended to maintain higher transpiration under prolonged drought. Interestingly, we observed a soybean-specific transpiration rate increase accompanied by phase shift under moderate soil drought. Time-series transcriptomic analysis suggested a dehydration avoidance mechanism of cowpea at early soil drought stage, in which the *VuHAI3* and *VuTIP2;3* genes were suggested to be involved. Multifactorial gene clustering analysis revealed different responsiveness of genes to drought, time of day and their interactions between the two crops, which involved species-dependent regulation of the circadian clock genes. Gene network analysis identified two co-expression modules each associated with transpiration rate in cowpea and soybean, including a pair of negatively correlated modules between species. Module hub genes, including the ABA-degrading gene *GmCYP707A4* and the trehalose-phosphatase/synthase gene *VuTPS9* were identified. Inter-modular network analysis revealed putative co-players of the hub genes. Transgenic analyses verified the role of *VuTPS9* in regulating transpiration rate under osmotic stresses. These findings propose that species-specific transcriptomic reprograming in leaves of the two crops suffering similar soil drought was not only a result of the different drought resistance level, but a cause of it.

## Introduction

By growing under environmental conditions with a wide range of variations in water availability, plants develop flexible water use strategies in response to water scarcity. As the gateway of both water efflux and CO_2_ intake, stomatal control is critical for balancing drought tolerance and growth under soil water shortages [1]. Some plants develop a sensitive response to drought at the stomata level, representing a conservative water use strategy; conversely, some other plants adopt a ‘profligate’ water use strategy that is marked by less sensitive stomatal control that allows transpiration of more water [2]. From an agronomic point of view, both strategies can be adaptive depending on the specific soil drought scenarios. Numerous studies have analysed water use traits in crops including legumes, grains, and trees [3–5], yet current mechanistic understanding of the trait plasticity is still limited.

Cross-species comparison of the dynamic water use traits is often challenged by the difficulty of generating homogenous progressive soil stress in a reasonably long term [6]. Recently, several high-throughput platforms have emerged, some being equipped with feedback irrigation systems, to enable precise control of soil water content in each unit [7, 8]. One of these platforms, the PlantArray, which is a lysimeter-based system, provides real-time monitoring of the parameters related to whole-plant water relations such as transpiration rate (Tr), growth rate, and water use efficiency (WUE) [8]. Species-dependent dynamic patterns of these traits in response to progressive soil stress have been delicately revealed in cowpea and tomato using PlantArray [9–11].

**Figure 1:**
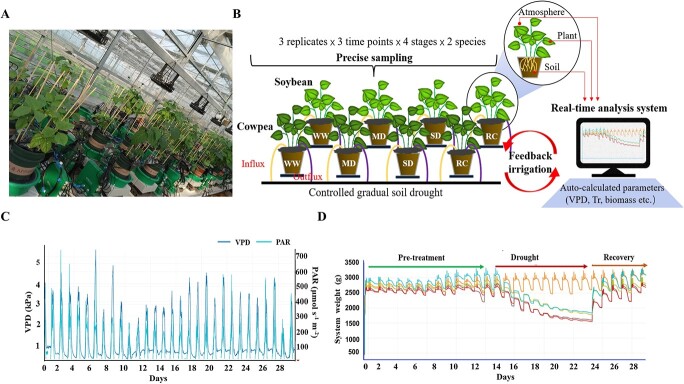
Overall experimental design. **A** and **B**, the lysimetric system where randomized experimental array consisting of multiple measuring units loaded with cowpea or soybean plants was set up. PlantArray combines gravimetric system, atmospheric and soil probes, irrigation valves, and controller in a unit. A real-time analysis system performs continuous and high-throughput statistical analysis for multiple sensors and sources (atmosphere, plant, and soil). The parameters related to whole-plant water relations such as dynamic vapor pressure deficiency (VPD), transpiration rate (Tr) and biomass were subsequently auto-calculated. Three plants grown in each pot and 12 pots of soybean or cowpea were set for phenotyping. Totally, 72 RNA-Seq libraries were constructed from leaf samples collected under various soil droughts. **C**, VPD and photosynthetically active radiation (PAR) conditions during the course of the experiment. **D**, Dynamics of system weight during the course of the experiment, which consisted of the pre-treatment, drought stress, and recovery phases.

Legumes are staple foods and important vegetables for many cultures worldwide [12]. Water deficiency at any stage, especially during the grain filling and reproductive phases, can affect legume plant growth and ultimately reduce yield and plant biomass [13]. The yield loss depends on the intensity and duration of drought, crop genotype and developmental stage [14]. Soybean (*Glycine max* L) native to East Asia is the most important legume crop globally, while cowpea (*Vigna unguiculata* L) indigenous to West Africa is one of the most drought-tolerant vegetable legumes popular in Asia [15, 16]. The two crops are known to exhibit different regulatory modes of water consumption when suffering drought stress, and have been frequently used in comparative studies of the shoot responses to soil water deficiency [2]. Cowpea typically demonstrates a slower rate of water loss and hence a higher leaf relative water content (RWC) and water potential than soybean as soil water is depleted, which is attributed to the better stomatal control [3, 17]. To date, the molecular mechanisms underlying the contrasting water use behaviors between the two species remain largely unknown.

In this study, we used the transpiration-interfaced automatic feedback irrigation function in PlantArray to generate consistent gradual soil drought for cowpea and vegetable soybean (known as maodou) plants. This enabled cross-species physiological and molecular comparisons in the shoot to be made under similar soil drought conditions while the ambient environments, including solar intensity and period, temperature and vapor pressure deficit (VPD), were identical. It also allowed for precise sampling of plant tissues under the intended soil water contents. Our results provide a high-resolution and comprehensive view on the physiological and molecular basis of the profligate versus conservative water use behaviors.

## Results

### Whole-plant water relations under progressive soil water deficit

**Figure 2:**
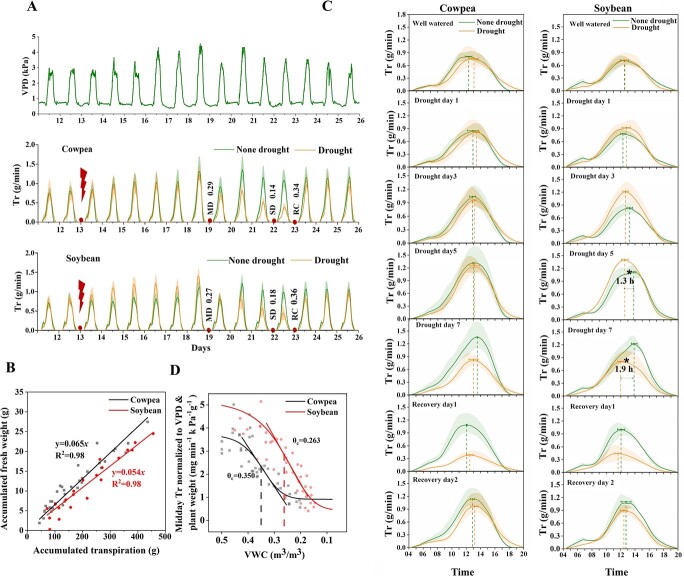
Whole plant water relations of soybean (‘ZN6’) and cowpea (‘TZ30’). **A**, Dynamics of transpiration rates (Tr) of the well-watered (WW) and drought-stressed cowpea and soybean plants. The date of the onset of irrigation reduction is marked with a flash icon and the VWCs (volumetric moisture content of soil) at the time of sampling in moderate soil drought (MD), severe soil drought (SD), and recovery (RC) stages are shown. **B**, Water use efficiency (WUE) of the two crops under well-watered condition. WUE was expressed as the amount of biomass produced per unit of water used by a plant. **C**, Daily Tr of the WW and stressed plants on specific days following treatments. Note the phase shift of midday Tr during MD in soybean. Asterisk indicates significant difference according to a *t*-test at the 5% level of significance. **D**, Plot of the midday Tr of the drought-stressed cowpea and soybean plants normalized to vapor pressure deficiency (VPD) and plant weight against VWC. θ_c_ represented the inflection point of soil VWC at which a plant showed the fastest decrease of Tr through stomatal control.

By deploying feedback irrigation to each pot according to the daily water loss (Table S1, see online supplementary material and see details in ‘Materials and methods’), we generated comparable strengths of gradual soil drought for cowpea and soybean plants and divided the treatment into four periods: the well-watered (WW), moderate soil drought (MD), severe soil drought (SD), and recovery phases (RC, Fig. 1). In both crops, the Tr exhibited a diurnal pattern with the maximum value being observed near noon (Fig. 2A). The WUE, which was expressed as the amount of biomass produced per unit of water used by a plant (g fresh weight g^−1^ water transpired), was greater in cowpea (0.065) than in soybean (0.054) under WW conditions, suggesting more efficient water use in the former (Fig. 2B). A comparison of the daily Tr between the WW and treatment groups showed no significant differences in cowpea during the initial days of irrigation reduction; in contrast, irrigation reduction had an immediate stimulatory effect on Tr in soybean that lasted for six days until the volumetric soil water content (VWC, the volume of water per unit volume of soil) reached a moderately low level of 0.27 (Fig. 2A, C). As drought progressed, Tr significantly decreased in both species. In soybean specifically, significant phase advancement of the midday Tr started to show on the fifth day of treatment (VWC = 0.35), and the extent of the phase shift increased with decreasing soil VWC (Fig. 2C). On the seventh day when the drought was severe (VWC = 0.18), the maximum phase change (1.9 hours) was observed (Fig. 2C). Following water resumption, the phase difference between the two groups rapidly disappeared. The phase difference of Tr was marginal in cowpeas in the whole course of the experiment.

Because Tr alone could not exclude the impact of canopy size and the environment, midday Tr (Tr_m_) between 12 pm and 2 pm was then normalized to VPD and plant weight. As shown in Fig. 2D, the daily Tr_m,VPD_ of each crop decreased gradually with the declining VWC, then fell down linearly, and ultimately approached the minimum. To describe the response curves of transpiration against water stress, we used a logistic function to fit Tr_m,VPD_ with VWC, where the maximum and minimum values of Tr_m,VPD_, the maximum decline rate of Tr_m,VPD_ and the corresponding critical VWC (θ_c_) determined the shape of the Tr_m,VPD_-VWC curves. Here, θ_c_ represented the inflection point of soil VWC at which a plant showed the fastest decrease of Tr through stomatal control. The results showed that under WW conditions, the Tr_m,VPD_ in cowpea reached a maximum rate of 3.747 ± 0.421 mg H_2_O min^−1^ kPa^−1^ g^−1^ fresh weight, which was lower than that of soybean (5.247 ± 0.818 mg H_2_O min^−1^ kPa^−1^ g^−1^ fresh weight), representing a more conservative water use strategy (Fig. 2D). The θ_c_ was much greater in cowpea (0.350 ± 0.017 m^3^/m^3^) than in soybean (0.263 ± 0.031 m^3^/m^3^), indicating that cowpea started constraining water loss under a more abundant soil water condition and corroborating it as a more conservative water consumer. Under very severe soil drought (VWC < 0.15), cowpea tended to maintain a higher level of midday Tr_m,VPD_ (0.913 ± 0.279 mg H_2_O min^−1^ kPa^−1^ g^−1^ fresh weight) than soybean (0.451 ± 0.205 mg H_2_O min^−1^ kPa^−1^ g^−1^ fresh weight, Fig. 2D). When irrigation was resumed, the Tr of both species recovered quickly to ~80% of the WW level within 2 days (Fig 2A).

### Overview of the transcriptomic data

To disclose the gene regulatory basis underlying water use behaviors, RNA-Seq was performed for cowpea and soybean leaves collected at WW, MD, SD, and the RC stages. At each sampling stage, the VWC was similar between the crops. A range of 38.2 to 70.6 million high-quality reads were generated from the 72 RNA-Seq libraries constructed from leaf samples collected under various VWCs (Table S2, see online supplementary material). High mapping rates (>92.4%) to the reference genomes were observed for all the libraries. Hierarchical cluster analysis showed high correlation among the biological replicates of each sample and a general trend of sample clustering more by time of day (TOD) than by treatment (Fig. S1, see online supplementary material). According to the Spearman correlation coefficients (SCC), VuMD-6&VuWW-6 (SCC = 0.972) and GmMD-6&GmRC-6 (SCC = 0.971) were the most correlated sample pairs in cowpea and soybean, respectively, while the VuMD-6&VuSD-16 (SCC = 0.313) and GmRC-6&GmMD-16 (SCC = 0.394) pairs were the least correlated. In general, GmMD-16 and VuSD-16 were the samples showing the least correlation to others in the two crops, respectively (Fig. S2A, see online supplementary material). TOD had a significant impact on both crops and showed complex interactions with the drought scenario (Fig. S2B, see online supplementary material). For example, the vast majority of cowpea differentially expressed genes (DEGs) were detected at midday under MD but late day under SD. In soybean, the least and most DEGs were recorded in the morning under MD and SD, respectively. A quantitative RT-PCR analysis of randomly selected genes confirmed the accuracy of the RNA-Seq data (Fig. S3, see online supplementary material).

### Transcriptomic reprogramming as revealed by pairwise comparisons

Pairwise comparisons between the samples collected at the same TOD in different treatment scenarios revealed a total of 4739 (15.9% of the 29 773 protein-coding genes) and 10 330 (19.5% of the 52 872 protein-coding genes) unique DEGs in cowpea and soybean, respectively (Fig. S2C, see online supplementary material). The top 20 DEGs were analysed for each of the nine pairwise comparisons in the two crops (Fig. S4, Table S3, see online supplementary material). Among them, the ABA signaling gene *HIGHLY ABA-INDUCED PP2C GENE 3* (*HAI3*) and the aquaporin gene *TONOPLAST INTRINSIC PROTEIN 2;3* (*TIP2;3*) drew our attention because they were only found in the top DEG list of cowpeas and were specific to the MD stage, thus were likely related to water conservation in cowpea under this condition, provided their putative functions in stomatal physiology [18, 19]. In soybean specifically, multiple *FASCICLIN-LIKE ARABINOGALACTAN* (*FLA*) genes, which are putatively involved in cell expansion and cell wall architecture [20], were among the top down-regulated DEGs. Two of soybean GABA transporter 1 encoding genes, which putatively play a role in regulating leaf senescence [21] were also among the top upregulated DEGs under both MD and SD.

To more comprehensively understand the functional relevance between all of the DEGs and water use behavior, gene ontology (GO) enrichment analyses were performed. In the leaves of MD-stressed soybean, GO terms related to photosynthesis, cell wall and fatty acid metabolism were enriched by down-regulated genes; in contrast, most of these GO terms were not enriched in cowpea leaves suffering from MD, which was consistent with a dehydration avoidance mechanism known for this crop (Fig. S5, see online supplementary material). As the soil drought became more severe, the two species exhibited more similar GO enrichment profiles, including those related to photosynthesis, cell wall, carbohydrate metabolism and protective/repair processes. Despite these commonalities, we observed interesting species-specific regulations. For example, GO terms related to phosphate/phosphorus/phosphorylation and lipid metabolism were enriched by upregulated genes only in soybean, indicating more profound involvement of morphological and nutritional factors such as cuticle and wax properties and Pi status in the soybean responses to severe drought. On the other hand, developmental process and aromatic compound biosynthetic process that is related to secondary metabolism were enriched in cowpea (Fig. S5, see online supplementary material), implying more versatile drought-coping strategies in this species.

### Differential expression of cowpea and soybean circadian clock genes in response to drought stresses

**Figure 3:**
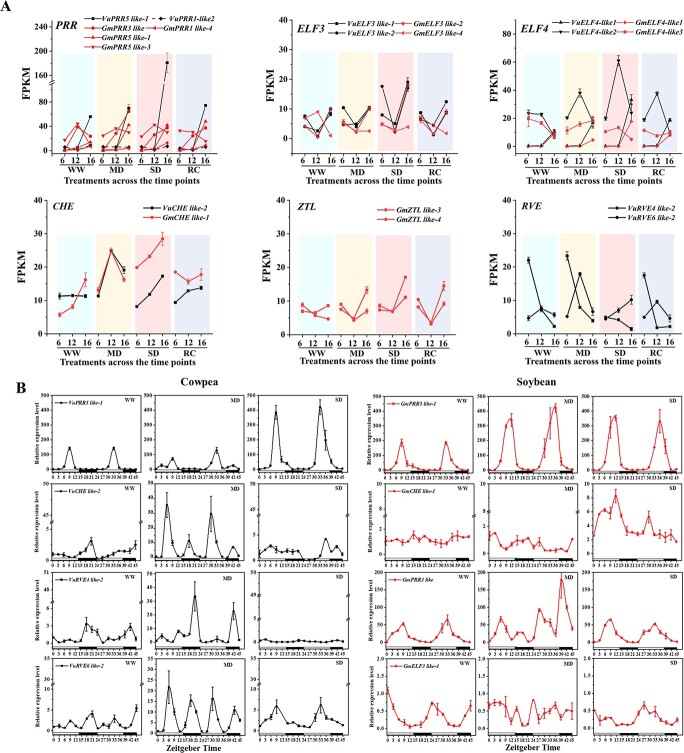
Drought affected the expressions of certain circadian clock genes in the two crops. **A**, the circadian clock genes showing altered expression levels in cowpea and soybean. These genes encode morning-phased RVE4 and RVE6, afternoon-phased PRR3, PRR5, and evening-phased ELF3, ELF4, PRR1, CHE, and ZTL. Genes were named according to the Arabidopsis orthologs as shown in Table S4 (see online supplementary material). **B**, higher-resolution gene expression analysis by qRT-PCR on cowpea and soybean leaves during WW, MD, and SD conditions. Timepoints are represented by Zeitgeber Time (ZT), starting from the time the light on (ZT0, at 6 am) and proceeding with 3 h intervals until ZT45. MD: moderate soil drought; RC: recovery phase; SD: severe soil drought; WW: well-watered control.

Since an interaction between TOD and drought scenario was indicated (Fig. S2B, see online supplementary material), we examined the dynamic expressions of the circadian clock genes to elucidate the effect of drought stress intensity on the transcript accumulation of core oscillators. A total of 32 and 44 putative circadian clock genes were identified from cowpea and soybean (Table S4, Fig. S6, see online supplementary material). The majority (56 out of the 76) of these genes kept their expression unchanged by drought treatment, while 20 genes showed expression alterations (Fig. 3A; Table S4, see online supplementary material). The expressions of cowpea *PSEUDO-RESPONSE REGULATOR 5 like-1* (*VuPRR5 like-1*), *EARLY FLOWERING 3 like-1 (VuELF3 like-1)*, and *ELF4 like-1* were sensitive to SD and less sensitive to MD at 4 pm. Their soybean counterparts including *GmPRR5 like-1*, *GmELF3 like-4,* and *GmELF4 like-3* significantly altered under MD and SD. The peak expression of *CCA1 HIKING EXPEDITION* (*CHE*) ortholog in cowpea occurred under MD, contrary to soybean *GmCHE* under SD. *REVEILLE* (*RVE*) and *PPR3* orthologs showed a species-dependent transcriptional regulation in cowpea and soybean (Fig. 3A). To validate the RNA-Seq results and enable understanding the effects of drought on diurnal expressions of the interested clock genes, RT-qPCR were then performed at the WW, MD, and SD stages, sampling for 2 days at a 3 h-interval. As shown in Fig. 3B, the investigated clock genes all exhibited a diurnal fluctuation in transcript enrichment under WW conditions, whereas MD and SD altered their expression patterns in varying degrees. MD did not much affect the phase and amplitude of *VuPRR5 like-1* in cowpea; in contrast, it significantly up-regulated the expression of *GmPRR5 like-1* and delayed its peak by three hours. *GmELF3 like-4*, the only gene showing decreased expression under both MD and SD in transcriptome data, showed arrhythmicity in MD but not SD in 2-day long expression. It is confirmed that *VuCHE like-1* and *GmCHE like-1* was more sensitive to MD and SD, respectively, and they also revealed differential diurnal patterns in two species. In addition, the *VuRVE4 like-2* and *VuRVE6 like-2* were obviously induced by MD, but they showed a single- and two-peak expression pattern, respectively. The expression of *GmPRR3* was significantly altered as a response to MD.

### Multi-factorial gene clustering analysis revealed species-specific gene regulations by drought, TOD, and their interactions

To comprehensively identify genes affected by TOD, drought, or their interactions, a generalized linear model (GLM)-based analysis of expression was then performed using *EdgeR* implementing custom codes (Data S1, see online supplementary material). We identified 5403 soybean genes as affected by drought, 651 by TOD, and 208 by their interactions. In cowpea, the numbers were 1766, 499, and 30, respectively (Table S5, see online supplementary material). The aforementioned genes were further clustered based on their expression modes using the k-means method. A total of 28 (12 by drought, 9 by TOD, and 7 by TOD × drought) and 39 (21 by drought, 12 by TOD, and 6 by TOD × drought) clusters were grouped in soybean and cowpea, respectively. Despite the fewer DEGs identified in cowpea as being responsive to drought or TOD × drought, they formed more or similar number of clusters than in soybean, suggesting higher heterogeneity of their expression regulations in cowpea. Functional annotations of each cluster were shown in Table S5 (see online supplementary material).

We were interested in gene clusters showing different expression patterns (*P* ≤ 0.05, Table S6, see online supplementary material) between MD and SD, because they may harbor the genes that were preferably expressed in either earlier or later stage of drought responses. When considering the significance of GO enrichment (FDR ≤ 0.05), the gene clusters with annotations in cowpea all fell into the drought-responsive category, while those in soybean fell into the TOD and TOD × drought-responsive categories as well. The cowpea gene clusters were functionally related to cell wall biogenesis, plastid, response to light stimulus, reproductive shoot system development, and ribosome (Fig. S7, see online supplementary material). In contrast, the soybean gene clusters were involved in oxidoreductase activity, cell cycle, cell periphery, transmembrane transporters, in addition to cell wall. Among them, the GO terms related to ion transport were enriched only in TOD and TOD × drought-responsive clusters. Clearly, the soil drought scenario and TOD interplayed in different ways in the two crops to affect the shoot gene expressions, which contributed to the formation of the contrasting water use behaviors (see discussions).

### Gene co-expression network and hub genes related to the transpiration rate

Through a weighted gene co-expression network analysis (WGCNA), we identified 17 and 21 gene modules in soybean and cowpea, respectively (Table S7, Figs S8 and Fig. S9A, see online supplementary material). Two co-expression modules each, viz. modules 9 and 17 (VuM9 and VuM17 hereafter) in cowpea and the modules 4 and 14 (GmM4 and GmM14 hereafter) in soybean, were significantly associated with Tr (*P* < 0.01), a key physiological trait related to water budgeting [22]. GO enrichment analysis revealed common terms in these four modules, such as plastid/chloroplast, responses to radiation and temperature, and responses to stress (Fig. S9B, see online supplementary material), which are known to relate to stomatal functions, thus corroborating the links of these modules with transpiration. We noted that VuM9 was enriched with many RNA/transcription-related GO terms, suggesting that the water conservation traits in cowpea leaves involved also active gene regulations, rather than merely a consequence of physiological drought avoidance. On the other hand, GmM14, but not VuM17, was enriched with the GO terms ‘endomembrane system’ and ‘endoplasmic reticulum’, despite the two modules both were primarily responsive to SD (Figs S9B and Fig. S10, see online supplementary material), implying a severely perturbed cellular homeostasis in soybean leaves [ 23]. Next, we interlinked the modules between the two organisms by measuring the correlation between their module eigengenes (MEs) using the WGCNA default ‘relating modules to external information’ analysis. This analysis found that, among the Tr-associated modules, the VuM9 was positively correlated with the GmM4 (r = 0.721, *P* = 0.008), while the VuM17 was negatively correlated with the GmM14 (r = −0.902, *P* = 5.89E^−5^). Notably, the GO term ‘response to heat’ fell into the two negatively correlated modules (see discussions).

**Figure 4:**
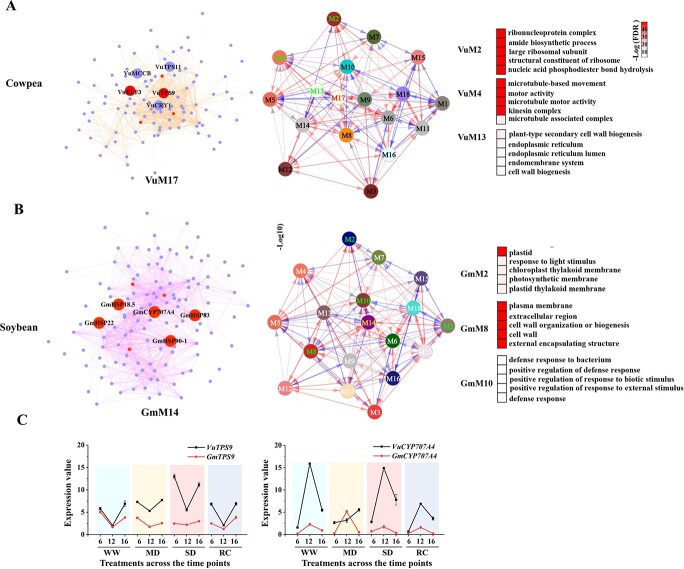
Weighted correlation network analysis. **A**, inter-modular network of the gene co-expression modules in cowpea (central panel), intra-modular gene network for the module VuM17 (left panel), and the functional annotations of the most interactive modules (VuM2, VuM4, VuM13) with VuM17. **B**, inter-modular network of the gene co-expression modules in soybean (central panel), intra-modular gene network for the module GmM14 (left panel), and the functional annotations of the most interactive modules (GmM2, GmM8, and GmM10) with GmM14. Only the top five enriched GO enrichments according to false discovery rate were shown. GmM1, which was also an interactive module with GmM14 and top-enriched with ‘plastid’, is not included in this figure because of the GO enrichment FDR values beyond the threshold (0.05). Red dots (left panels in **A** and **B**) denote hub genes; medium purple dots (left panel in **A** and **B**) denote other genes in the network of module VuM17 or GmM14. Arrowed lines in blue and red color (central panels in **A** and **B**) denote positive and negative interactions, respectively. **C**, Dynamic expression patterns of the *CYP707A4* and *TPS9* orthologous genes in the two species under various drought scenarios. MD: moderate soil drought; RC: recovery phase; SD: severe soil drought; WW: well-watered control. 6, 12, 16 denote time of day (6 am, 12 pm and 4 pm).

By using the criteria of intramodular connectivity (*K*_ME_) ≥0.85 and absolute gene significance (GS) ≥0.85, we identified five and eight hub genes from VuM17 and VuM9, respectively (Fig. 4A and B; Table S7, see online supplementary material). From GmM4 and GmM14, we identified 26 and 9 hub genes, respectively (Table S7it should be Table S8, see online supplementary material). The soybean hub genes included those encoding various types of transcription factors, heat shock proteins, transporters, and noticeably, the ABA-degrading enzyme abscisic acid 8′-hydroxylase 4 (*CYP707A4*) [24]. The *GmCYP707A4* was transcriptionally upregulated at noon under MD and had a positive GS value, indicating that its expression increased Tr; however, its ortholog in cowpea was down-regulated and less abundantly expressed under the same stress despite its higher expression under the WW and SD conditions (Fig. 4C). Given the close relationship between ABA and stomatal closure, the higher expression of *GmCYP707A4* under MD is postulated to relate to the more profligate water use trait in soybean leaves under this specific soil drought scenario. The hub genes of cowpea included those encoding the ABA-signaling component phosphatase 2C family protein, trehalose-phosphatase/synthase 9 (TPS9), RAB GTPase homolog A6B protein, far-red elongated hypocotyl 1 and so on. Among them, *VuTPS9* (*Vigun02g076000*) aroused our particular interest (Fig. 4A), because trehalose has emerged as an important singling molecule in stress responses [25, 26]. The expression of *VuTPS9* showed an obvious fluctuation across a day, and was higher under MD and SD than WW. In contrast, the soybean ortholog of *TPS9* (*Glyma.02G033500*) showed rather stable expression over the course of gradual drought (Fig. 4C). Intra-modular gene network further showed that *VuTPS9* interacted, at the highest weight, with the genes encoding TPS11, ELF3, the energy-related 3-methylcrotonyl-CoA carboxylase (MCCB), and circadian-related cryptochrome 1 (CRY1) (Fig. 4A; Table S8, see online supplementary material).

### Functional validation of *VuTPS9* for its role in regulating transpiration rate (Tr)

**Figure 5:**
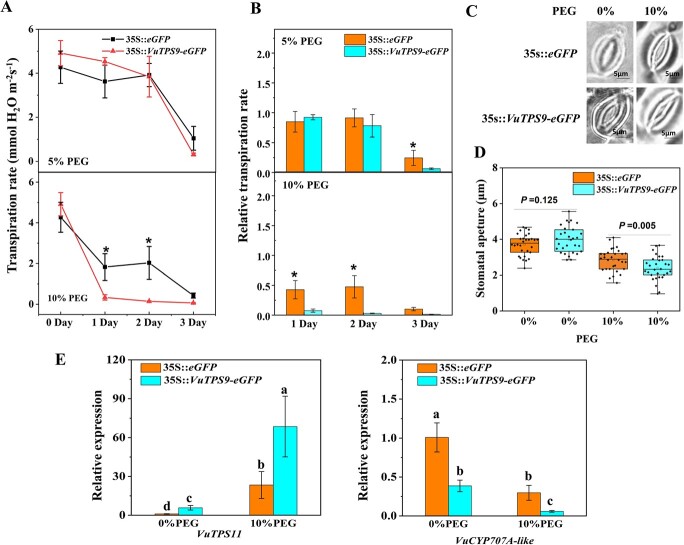
The effect of *VuTPS9* overexpression on transpiration rate and stomatal closure. **A** and **B**, the transpiration rates (Tr, A) and relative Tr (B) in the leaves of *VuTPS9*-OE (35S::*VuTPS9-*eGFP) and 35S::*eGFP* lines after PEG-6000 treatment at different concentrations for 0, 1, 2, and 3 days. To calculate the relative Gs, the average Tr amount on day 0 of each line was set as 1. Data are means of at least three biological replicates and are shown with vertical error bars (±SD). Asterisk indicates significant differences according to a *t*-test at the 5% level of significance. **C**, representative stomatal images of the two lines exposed to 10% PEG-6000 or mock (water) treatments. **D**, stomatal aperture values. Dots represent data measured from 30 stomata under each treatment. *P*-values were calculated from a *t*-test. **E**, relative expressions of *VuCYP707A-like* (*Vigun03g186900*) and *VuTPS11* (*Vigun03g378000*) in leaves of *VuTPS9*-OE and 35S::*eGFP* lines after PEG-6000 treatment for 1 day. Different letters indicate significant differences according to a Tukey’s test at 5% level.

Among the aforementioned hub genes, *TPS9* and its orthologs had not been functionally characterized in any plant species. Moreover, the cowpea and soybean *TPS9* orthologs exhibited different regulatory patterns under drought treatment, *VuTPS9* was hence selected for functional validation. Gene family analysis identified 10 *TPS* genes from the cowpea genome, and the phylogenetic tree suggested that *VuTPS9* belongs to the Class II subfamily (Fig. S11, see online supplementary material). Here, 5% and 10% PEG-6000 treatments were adopted to mimic the different strengths of osmotic stress. The Tr and stomatal conductance (Gs) of leaves were compared between the transient transgenic lines of 35S::*VuTPS9-eGFP* (*VuTPS9*-OE) and 35S::*eGFP* overexpression lines, which showed no significant difference before treatment (Fig. 5A; Fig. S12, see online supplementary material). Treatment with 5% PEG reduced Tr and Gs to a similar level in the two lines on each of the three days during the experiment; 10% PEG treatment caused a rapid and sharper decline in Tr and Gs in both lines, particularly in the *VuTPS9*-OE plants (Fig. 5A; Fig. S12, see online supplementary material). To better display the daily inhibitory effects in the two lines, the relative Tr and Gs to the 0-day value were calculated. As shown in Fig 5B and Fig. S11C, on the first day of 10% PEG treatment*,* the levels of Tr and Gs decreased by 92% and 95%, respectively, in the *VuTPS9*-OE plants, as opposed to 57% and 73% in the 35S::*eGFP* line. Consistent with this phenotype, the stomatal aperture in the former line was more sensitive than that in the latter line, as measured 1 day after 10% PEG treatment (Fig. 5C and D).

Lastly, to test the hypothesized co-players of *VuTPS9* based on WGCNA, we examined the expressions of the aforementioned *VuTPS11* (*Vigun03g378000*), which was co-expressed with *VuTPS9* in the module VuM17, as well as *VuCYP707A-like* (*Vigun03g186900*), which was a member of the VuM17-interacting module VuM2. These two genes were selected because they also were putatively involved in trehalose signaling and ABA degrading, respectively. We found that the expressions of both genes were indeed altered in leaves of the *VuTPS9*-OE plants relative to the control line. Expression of *VuTPS11* was enhanced by *VuTPS9* overexpression and 10% PEG treatment, while an opposite trend was observed for *VuCYP707A-like* (Fig. 5E). Therefore, trehalose and ABA signaling appeared to interplay to confer the water use behavior in cowpea.

## Discussion

Transpiration-interfaced automatic feedback irrigation based on soil VWC could accurately control the speed of soil water depletion in each pot, thus creating highly comparable drought scenarios in different pots. This approach reduces the confounding effects of leaf size and growth rate [17] and maximizes the likelihood of finding genotypic drought response differences. The whole plant transpiration-based assay avoids problems of upscaling conventional leaf-based traits, such as photosynthetic carbon isotope discrimination, to the plant or field scale [27]. The term ‘physiolomics’ was recently proposed to refer to this emerging subject of high-throughput physiology-based phenotyping [28]. Benefiting from the long-term phenotyping and modeling of Tr_m,VPD_, we discovered that cowpea had the potential to maintain a higher transpiration rate under very severe soil drought (VWC < 0.15). This finding is reminiscent of an earlier study reporting lower lethal RWC values of leaves in cowpea (40%) than in soybean (50%) [29], and argued that conservation or profligation in water use is a conditional concept. Given the viewpoint that any plant traits to avoid or postpone drought become ineffective under terminal drought or in soils with very low water-holding capacity [2], we propose that the water regime-dependent conservative/profligate water use behavior in a plant is an adaptive trait to help balance survival and productivity under dynamic environmental changes. The high resolution and continuity of the physiolomic assay also led to the detection of the soybean-specific short-term increase along with a phase change of Tr_m_ under MD conditions. Making a conclusive interpretation to this phenomenon is still difficult at present, but it might be due to an interplay between MD and midday high temperature. Tr is known to be related to canopy temperature [30], and soybean is more heat sensitive than cowpea [15, 31]. Our assumption could find some support from the molecular data that orthologs of the clock gene *ELF3*, which is also a thermal responsiveness component [32], showed different regulations by drought between cowpea and soybean. WGCNA further uncovered a negative correlation of two Tr-associated co-expression modules comprising the GO term ‘response to heat’ between the two crops, reinforcing that heat responses may underpin the phase change of Tr_m_.

The soil drought signal perceived by the roots triggers the accumulation of ABA and the initiation of the ABA signaling cascade, which is key to the induction of stomatal closure in shoots [18, 33]. The hormonal action of ABA is precisely controlled by its biosynthesis, catabolism and transduction [34]. We found that the ABA signaling repressor gene *HAI3* was among the top-upregulated genes in cowpea only, and the ABA-degrading gene *CYP707A4* was identified as a soybean-specific hub gene associated with Tr. More interestingly, the regulation of both genes showed a drought scenario-dependent feature. We therefore postulate that the more profound and earlier-stage activation of *HAI3* in cowpea contributed to the higher sensitivity of stomatal control and hence drought avoidance under MD, while the MD-specific upregulation of *CYP707A4* in soybean is likely part of the fine-tuning mechanism to maintain higher Gs through ABA degradation. Taken together, the results suggest a species-dependent subtle regulation of ABA signaling via the balance of ABA signal activation and ABA degradation.

Trehalose, a soluble sugar synthesized via a two-step reaction involving trehalose-6-phosphate synthase (TPS), is known for its role in metabolic and osmotic regulation in a variety of organisms. Recently, the signaling role of trehalose has emerged in plants. Arabidopsis *tps1* and *tps5* (Class II) loss-of function mutants showed insensitivity and hypersensitivity to ABA induction of stomatal closure, respectively, suggesting that the two genes exerted contrasting roles in the adjustment of stomatal aperture under stressed conditions [35, 36]. Here, we identified *VuTPS9* belonging to the class II subfamily as one of the hub genes and revealed the different responses of the *TPS9* orthologs to progressive drought between cowpea and soybean. We provided evidence that overexpression of *VuTPS9* increased osmotic stress-induced Gs and Tr inhibition under drought conditions. This effect is similar to that reported for *AtTPS1*, a class I *TPS* gene, but contrasting to that of the class II gene *AtTPS5*, arguing that the biological functions of *TPS* genes may not necessarily be related to their subfamily classifications. Our network analysis further suggests that the trehalose signal works with the circadian signal, ABA and energy metabolism processes to regulate transpiration rate in the shoots under soil drought conditions. Put together, these results add to our knowledge on the roles of the *TPS9* gene in drought stress responses.

**Figure 6:**
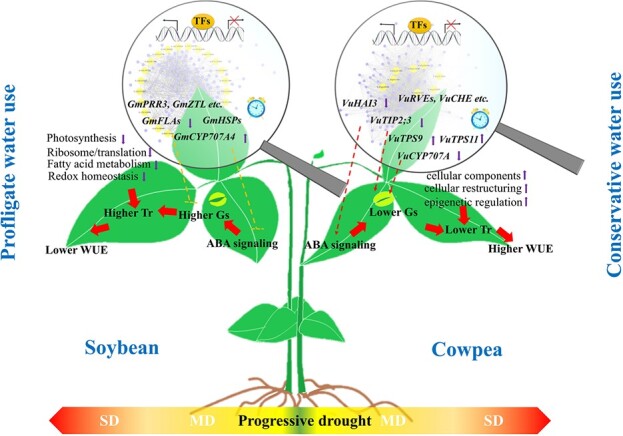
A proposed model of mechanism underpinning the contrasting water use strategies in soybean and cowpea. The involvement of genes related to hormonal, physiological, and structural regulations such as *VuHAI3*, *VuTIP2;3*, *VuTPS9*, *GmCYP707A4*, *GmFLAs*, and their putative interactive genes such as *VuRVE*, *VuCRY1*, *VuTPS11*, are highlighted. Arrowheads denote the effect of activation and blunt ends denote the effect of inhibition. Up and down arrows indicate up- and down-regulations, respectively. Dotted lines indicate that confirmation is required. Gs: stomatal conductance; MD: moderate soil drought; SD: severe soil drought; TFs: transcription factors; Tr: transpiration rate.

The endogenous circadian clock, a time-keeping mechanism, confers environmental fitness to higher plants. The reciprocal interaction between clock- and drought-responsive genes has been reported. For example, soybean orthologs of the morning loop component CCA1/LHY were found to negatively control drought tolerance by gating ABA biosynthesis and ABA signaling [37]. A prevailing transcriptomic reconfiguration by drought stress was observed in the late day in *poplus* [38]. Our results revealed different sensitivities as well as patterns of amplitude and phase of certain clock genes to soil drought between cowpea and soybean. Similarly, Li *et al.* (2019) [39] reported that the interfaces between the soybean clock and abiotic stress signals were quite different from those in Arabidopsis, suggesting the contribution of species-specific circadian phases in response to environmental cues. The phase shift of gene expression can have a profound effect on related biological processes, as demonstrated in the case of iron utilization efficiency in soybean [36]. In this study, MD significantly delayed the peak of *GmPRR5 like-1* but had little impact on *VuPRR5 like-1*. In addition, the expressions of two *RVE* orthologs in cowpea but not in soybean showed alterations under MD and SD. These results suggested that the core oscillators regulate water budgeting in legumes. Furthermore, prevailing transcriptomic reconfiguration was observed in the late afternoon, which was evident at the MD stage for soybean, compared to the SD stage for cowpea. The elevated expression of clock genes in late days has been related to increased ABA synthesis in barley [40]. Given the conserved relationship between ABA and drought response in plants, we assume that the interplay among crop type, drought scenario and TOD had an impact on ABA accumulation and was partly accountable to the contrasting water use behaviors in the two crops. Recently, the influences of the circadian clock on the long-term WUE of Arabidopsis were reported [41], providing evidence of the circadian clock gating of plant water budgeting.

Collectively, a model depicting the gene regulatory mechanisms underlying the differential water use strategies is proposed (Fig. 6). In cowpea, *VuHAI3*, a negative regulator of ABA signaling is down-regulated under MD, thus may quickly trigger ABA signaling for water conservation. The down-regulated *VuTIP2;3* and up-regulated *VuTPS9* are proposed to accelerate drought-induced stomatal closure, alongside the roles of many other genes including *VuTPS11* and *VuCYP707A*. Up-regulated genes related to cellular components may modulate water use efficiency via cellular re-structuring and epigenetic regulation. In soybean, the higher Tr under MD caused by greater Gs is likely related to the high expression of the ABA degradation enzyme *GmCYP707A4*, accompanied by gene expression changes related to photosynthesis, leaf senescence, ribosome/translation, fatty acid metabolism and redox homeostasis. Circadian clock gene such as *PRR5* and *CHE* also play an important role in modulating water use strategies by reshaping the rhythmic patterns, in a species-specific and/or drought strength-dependent manner. In the future, a more comprehensive understanding can be achieved by combining physiological phenotyping with morphological phenotyping.

## Materials and methods

### Plant materials and irrigation conditions

Plant materials include one cowpea (‘TZ30’) and one soybean (‘ZN6’) genotype, respectively. The physiological experiment was conducted using the ‘PlantArray’ (Plant-DiTech, Yavne, Israel) phenotyping platform that installed in a greenhouse in October to November 2019 in Huai’an (33.62^°^N, 119.02°E), China (Fig. 1). PlantArray combines gravimetric system, atmospheric and soil probes, irrigation valves and controller in a unit [8]. Four-week-old plants were transferred into the load cells on the system, with three plants grown in each pot and 12 pots set for each species. Each pot was filled with 3.9-L vermiculite and nutrient soil mixed in a 2:1 (v:v) ratio whose surface was wrapped with plastic film to prevent evaporation, and was irrigated by multi-outlet dripper assemblies (Netafim, Aviv-Yafo, Israel) that were pushed into the soil and connected to an automated feedback system as described previously [8, 10]. Before the start of the experiment, all units were calibrated for reading accuracy and drift levels under constant load weights. The experiment lasted for 28 days and the drought treatment started on day 13, during which gradual deficit irrigation was given during night by the feedback irrigation system that reduced the irrigation levels every day for each pot based on the daily water loss (Table S1, see online supplementary material). This approach enabled dynamically comparable soil drought strength (feedback drought slope) to be imposed between the two species over time, despite their heterogenous growth rates and plant sizes. Note that measuring leaf area is no longer necessary here with PlantArray for normalized (to biomass) plants [8]. Physiological profiles of the plants in each pot were compiled automatically during the whole experiment which was divided into four treatment periods: WW, MD, SD, and RC. The soil–plant-atmosphere parameters including VPD, photosynthetically active radiation and temperature were monitored simultaneously and continuously with a high resolution (every 3 minutes).

### Data acquisition and analysis of the water relations parameters

Data acquisition were previously described in details by Halperin *et al.* (2017) [8]. Briefly, the daily whole-plant transpiration was calculated by the difference of measured system weight at pre-dawn and evening. The daily cumulative biomass gain and transpiration throughout a 7-day well-irrigated period was fitted by a linear function, where the slope was determined as the WUE. The plant weight was calculated as the sum of the multiplication of the cumulative transpiration during the period by the WUE and the initial plant weight. The latter, determined as the difference between the total system weight and the sum of the tare weight of pot plus drainage container, weight of soil at pot capacity, and weight of water in the drainage container at the end of the free drainage.

The momentary whole-plant Tr was computed by multiplying the first derivative of the measured system weight by −1. The average whole-plant Tr at mid-day normalized to VPD (Tr_m,VPD_, between 12 am and 4 pm) and the corresponding volumetric moisture content of soil (VWC) were fitted well with a 4-parameter logistic function as the following,

$$ \begin{align*} {\mathrm{Tr}}_{\mathrm{m},\mathrm{VPD}}=\frac{\mathrm{A}1-\mathrm{A}2}{1+{\left(\frac{\mathrm{VWC}}{{\mathrm{VWC}}_0}\right)}^p}+A2 \end{align*}$$

where A1 and A2 were the maximum and minimum values of Tr_m,VPD_ depending on the fitting result at the well-irrigation period and severe-drought period, respectively. *p* indicates the maximum slop of the curve at the inflection point, ie., VWC_0_, where *p* and VWC_0_ mainly determines the sensitivity of Tr_m,VPD_ in response to VWC.

### RNA isolation and RNA-Seq

One leaflet each from the same trifoliate leaves of the three seedlings in a pot was collected and pooled, at 6 am, 12 pm and 4 pm of the sampling day, respectively. Three biological replicates were analysed per sample. RNA was extracted using Trizol reagent (Invitrogen, Carlsbad, CA, USA). Five micrograms of total RNA from each sample were used to construct an RNA-Seq library by using the TruSeq RNA Sample Preparation Kit according to the manufacturer’s instructions (Illumina, San Diego, CA, USA). A total of 72 libraries (Table S2, see online supplementary material) were constructed and then sequenced on the Novaseq 6000 platform.

### Raw data processing and pairwise differential expression analysis

The raw RNA-Seq reads were filtered and trimmed using SeqPrep (https://github. com/jstjohn/SeqPrep) and Sickle (https://github.com/najoshi/sickle) with default parameters. The cleaned reads were combined and aligned to the soybean reference genome WM82.a4.v1 (Phytozome v13) or the cowpea reference genome *V. unguiculata* v1.1 [42] using TopHat (V2.1.0). The count of the mapped reads from each sample was derived and normalized to fragments per kilobase of transcript length per million mapped reads (FPKM) for each predicted transcript using Cufflinks (v2.2.1).

Pairwise comparisons were made between samples collected at the same timepoint for each crop. To reduce noises, only genes having an FPKM ≥1 in the three replicates of one or both samples in a comparison and a coefficient of variant (CV) < 0.2 among the three replicates were considered. The genes exhibiting a difference of at least twofold change with the false discovery rate (*q*-value) ≤0.05 were considered as DEGs.

### GLM-based differential expression analysis and clustering of the DEGs

Let y*_i_* = (y*_e_* (1), y*_i2_* (2), ..., y*_ik_*(*t*)) denote the expression measure of gene *i* at different time points of day under treatment *k*. Thus, the GLM that determines the gene expression of gene *i* is expressed as follows:

(1) \begin{align*} Y_{i}=\mu+\alpha T+\beta T +\lambda TK+\varepsilon i, T=1,\ldots,t; K=1,\ldots,k, \end{align*}

where *μ* is the mean value; *T* is the time vector; *K* is the indicator variables that describe the different treatments; *α* and *β* are the coefficients of time and treatment; *λ* reflects the interaction between time and treatment; and *εi* is the random error, which is generated from a negative binomial distribution. Here, we implemented model (1) for DEGs screening by *edgeR* package in the R platform [43]. We used three-hypothesis tests to detect genes that were differentially expressed in response to the different factors including drought treatment, TOD, and the drought × TOD interaction. An FDR value of 0.05 was adopted as the threshold for significance.

The k-means method was used to cluster the DEGs into distinct groups. Here, the |log_2_FC| value was used as an additional standard to screen the DEGs for higher stringency. The |log_2_FC| threshold was set to 1, 0.5, and 0.25 for the drought, TOD, and drought × TOD category, respectively, by considering the nature of log_2_FC calculation in the model. We applied the between-cluster sum of squares to determine an optimal number of gene groups, using 0.75 as the threshold value. The *t*-test was used to identify whether a significant difference existed between a pair of clusters from different combinations.

### Gene ontology (GO) enrichment analysis

GO enrichments were analysed for the DEGs using AgriGO V2.0 [44] under a *q*-value threshold of 0.01 for statistical significance. The cowpea reference (787 759 GO assignments for 14 079 genes, 56 assignments/gene) was comparable to that of soybean (1 444 608 GO assignments for 25 073 genes, 58 assignments/gene) in AgriGO V2.0.

### Circadian clock gene analysis

To identify putative circadian clock genes from soybean and cowpea, the sequences of 14 known Arabidopsis core clock proteins [45] were used as queries in BLAST searches against the soybean and cowpea genomes (Table S4, see online supplementary material). An *e*-value cutoff of 1*e*^−20^ was applied and the most similar sequence for each BLAST search was selected. The expression levels of the identified clock genes as represented by FPKM were extracted from RNA-seq data. For higher-resolution expression analysis, qRT-PCR was performed with plants cultivated in a growth room. Three seedlings each of cowpea (‘TZ30’) or soybean (‘ZN6’) were cultivated in a pot with 3.9-L vermiculite and nutrient soil mixed in a 2:1 (v:v) ratio, and a total of 48 pots were set for each species. The ambient conditions were: 16 h light/8 h night cycles, 1000 μmol m^−2^ s^−1^ of white light, 28°C, and a humidity of 85%. When the seedlings were 4 weeks old, irrigation was withheld to impose progressive drought stress. The VWC of each pot was determined using 5TE soil moisture sensors (Decagon Devices, Inc., pull man, WA, USA), whose ranges for sampling were: 0.38 ~ 0.42 (WW), 0.22 ~ 0.27 (MD), and 0.10 ~ 0.15 (SD) treatment. Fully expanded leaflets were collected from the seedlings under WW, WD, and SD stages, each at a 3-h interval from the time the lights came on (Zeitgeiber Time 0, ZT 0). Three biological replicates were included in sampling.

### Weighted gene co-expression network analysis

The co-expression gene network modules were inferred from DEGs using a WGCNA implemented in R. FPKM values of the three replicates were averaged and log transformed. The automatic one-step network construction and module detection method with default settings were used, which include an unsigned type of topological overlap matrix (TOM), a power *β* of 10, a minimal module size of 30, and a branch merge cut height of 0.25. Modules were determined by the dynamic tree cut method. The module eigengene (ME) value, which is defined as the first principal component of a gene module [46], was calculated and used to test the association of the modules with drought treatment. Spearman correlation between each consensus ME and Tr was calculated to find modules of interest. Lasso regression analysis implemented in the R package ‘glmnet’ was used to construct the inter-modular network through a custom script (Data S1, see online supplementary material).

### Quantitative RT-PCR

qPCR was performed on qTOWER®3 Real-Time PCR detection system (Analytik Jena, Tostedt, Germany) using TOROGGreen® qPCR Master Mix (Toroivd, Shaihai, China). Quantification of relative expression level was achieved by normalization against the transcripts of house-keeping genes [47] *VuACTIN*, *VuELF1a*, and *Gmβ-ACTIN*. The primer sequences are listed in Table S10 (see online supplementary material).

### Analysis of *VuTPS* genes

The complete protein sequences of TPS of Arabidopsis, rice, and soybean were downloaded from Phytozome (https://phytozome-next.jgi.doe.gov/). They were then subjected to BLASTP searches against the cowpea genome (version 1.2). The putative TPS proteins were further confirmed with the conserved domain search tool SMART. In total, 10 putative TPS genes were identified in cowpea. The phylogeny was constructed by using the maximum-likelihood method in MEGA6.0 with 1000 bootstrap replicates.

### Transient overexpression assay

The full cDNA fragment of *VuTPS9* amplified by the primers (F:5′-CGACGACAAGACCGTCACCATGGCGTCAGGATCATATGC-3′; R:5′-GAGGAGAAGAGCCGTCGAGCCATGCTTTCAAAGGAAACT −3′) was cloned into the pVC vector fused with the eGFP sequence at the 3′-terminus. The resulting CaMV 35S::*VuTPS9-eGFP* construct was introduced into *Agrobacterium rhizogenes* strain *GV3101*. Agrobacterium culture containing the construct was resuspended in infiltration buffer (10 mM MES-KOH, pH 5.6, 10 mM MgCl_2_ and 100 μM acetosyringone) and adjusted to a concentration of OD_600_ = 1.2. The suspensions were infiltrated into the fully developed unifoliate leaves of 10-day-old cowpeas grown in Hogland solution. After one day of transformation, the seedlings were exposed to Hogland solution supplemented with 5% or 10% PEG-6000 for 3 days. The expression of *VuTPS9-eGFP* was determined by the observation of eGFP signals.

### Portable device-based measurement of stomatal conductance (Gs) and transpiration rate (Tr)

The Gs and Tr of the transiently transformed leaves were measured using an infrared gas analyser-based portable photosynthesis system (LI-6400; Li-Cor, Lincoln, NE, USA) mounted with a red/blue LED light source. The environmental condition was maintained at a temperature of 28°C, humidity of 85% and the PPFD of 1000 μmol m^−2^ s^−1^. The measurements were made at midday (12 pm to 1 pm) on each day after PEG treatment. Data were acquired from five biological replicates of the control and *VuTPS9-OE* plants.

## Acknowledgments

We thank Wenzhao Xu and Dexu Luo for assistance in the greenhouse experiment, Liang Zeng for assistance in bioinformatical analyses and Menachem Moshelion and Amir Mayo for assistance in running PlantArray. This work is supported by the National Key Research and Development Program of China (Grant No. 2022YFE0198000), National Natural Science Foundation of China (Grant No. 31772299, 31861143044), and the Natural Science Foundation of Zhejiang Province (Grant No. LQ21C150004).

## Author contributions

P.X. designed the research and wrote the manuscript. P.F. participated in the experiments, data analysis, and wrote the manuscript. T.S. and A.K.P. participated in high-throughput physiological phenotyping analysis. P.F., L.J., and S.C. performed the transcriptomic data analysis. X.W. and Y.H. performed the transient overexpression assay. M.L. participated in the measurement of Tr and Gs.

## Data availability

The RNA-seq raw data are accessible in GenBank with the accession numbers stored in Table S11 (see online supplementary material). The R codes for the GLM, clustering, and inter-modular network analyses are available in Data S1 (see online supplementary material).

## Conflict of interests

The authors declare that they have no conflict of interest.

## Supplementary data

Supplementary data is available at *Horticulture Research* online.

## Supplementary Material

Web_Material_uhac287
