# Supplementary material for: Understanding water conservation vs. profligation traits in vegetable legumes through a physio-transcriptomic-functional approach
Source: Hortic Res. 2022 Dec 29;10(3):uhac287. doi: 10.1093/hr/uhac287 (PMC10015340; doi:10.1093/hr/uhac287)
Supplement: Web_Material_uhac287 [file web_material_uhac287.zip › Table S10.docx]

**Supplemental Table S****10. Primer sequences used for qRT-PCR analysis**

| **Accession number** | **Gene name** | **Forward primer** | **Reverse Primer** |
| --- | --- | --- | --- |
| *Vigun05g248600* |  | 5’-CACTTGGCTACAATCCCTATCA -3’ | 5’-ACGGTCCAACCCAGTAAATC-3’ |
| *Vigun05g282700* |  | 5’-CACTGGTCTCTTCTACTGGATTG-3’ | 5’-GCAACACCGTGGATAGGAATA-3’ |
| *Vigun09g276100* | *VuTPS6* | 5’-CCGTCATCGGTCACAAGAAA-3’ | 5’-GTGCAGTAGACGAGAACGAAG-3’ |
| *Vigun06g141300* |  | 5’-CCATTGTTCGTGAAGTCGTGAG-3’ | 5’-GTGAGTTGTTGTTCGGCAGAG-3’ |
| *Vigun10g181500* |  | 5’-TCTCTGGTGGGTCGATGAA-3’ | 5’-CAATAAGAGGTCCAACCCAGTAG-3’ |
| *Vigun04g203000* | *VuACTIN* | 5’-TCAGGTGTCCAGAGGTGTTGTA-3’ | 5’-ATGGTTGTGCCTCCTGAAAGTA-3’ |
| *Vigun03g378000* | *VuTPS11* | 5’-AAGTATTGCTCGTTCGGTGTCTA-3’ | 5’-TGGATGAGGATGGTGTTGATGG-3’ |
| *Vigun03g186900* | *VuCYP707A-like* | 5’-ACTAGGTTGTCCATGCGTGAT-3’ | 5’-CTGATGCGACTGTGGTATTCTC-3’ |
| *Vigun04g088900* | *VuEF1A* | 5’-GCCTGGTATGGTGGTGACTT-3’ | 5’-GCGAACTTCACTGCAATGTG-3’ |
| *Vigun09g140300* | *VuPRR5 like-1* | 5’-AGAGAAGTCAGTGAGAGTGGAGA-3’ | 5’-GCTATAATCTGCCTTGTTGAGTCAT-3’ |
| *Vigun01g156500* | *VuREV4 like-2* | 5’CTGAGGAGGAGCATGACAAGTT-3’ | 5’-TTAGGACGAGGAGGAGGAACAT-3’ |
| *Vigun07g222100* | *VuREV6 like-2* | 5’GCAGTAACAGTGGAAGTCCTAGA-3’ | 5’-ACAGTAGCACAGTCTCAACATCT-3’ |
| *Vigun02g007600* | *VuCHE like-2* | 5’CACCAACACCAGCACCATC-3’ | 5’-GACAACGAGGCGAGCAAAT-3’ |
| *Glyma.04g003200* |  | 5’-CATCACTGGTACCGGGATTAAC-3’ | 5’-CGAATGGTCCAACCCAGAATA-3’ |
| *Glyma.04g083200* |  | 5’-GCCGCAACAGCTTCTTATCT-3’ | 5’-CCCAAACTACTCCTTGACCATAC-3’ |
| *Glyma.08g015300* |  | 5’-ACTGTCATGGGTGTCAACAG-3’ | 5’-GCAGTAGACAAGGGCAAAGA-3’ |
| *Glyma.13g224900* |  | 5’-TCCCTTCCGGTTCTGATTTG-3’ | 5’-GCTCACCAATCGCTCTGTTA-3’ |
| *Glyma.19g181300* |  | 5’-CTAGGGCCTGCTGTCATATTC-3’ | 5’-CACGGACTGGTGGTAGAATG-3’ |
| *Glyma.02g091900* | *GmACTIN* | 5’-TCAGCCACACTGTCCCTATC-3’ | 5’-GCTCGTAGTCAAGGGCAATG-3’ |
| *Glyma.15g050200* | *Gmβ-ACTIN* | 5’-GAGCTATGAATTGCCTGATGG-3’ | 5’-CGTTTCATGAATTCCAGTAGC-3’ |
| *Glyma.06g136600* | *GmPRR5 like-1* | 5’-GACAATCATCAACCACAGGCATT-3’ | 5’-GCTTCACAGTCAGGCTTCGTA-3’ |
| *Glyma.12g073900* | *GmPRR3 like* | 5’-TCAAGTTGGCAGTCCTCATCC-3’ | 5’-CAAGATCAATAAGTTCATGGCACTC-3’ |
| *Glyma.08g197500* | *GmELF3 like-4* | 5’-GCAAGGTAGCAGTGTCCGTA-3’ | 5’-TGTCTCCTCATCTTCATTCCTGTT-3’ |
| *Glyma.10g285900* | *GmCHE like-1* | 5’-CTCTCCCACTCCCTTCATCCT-3’ | 5’-CTGCGACACCGACACCATT-3’ |
|  |  |  |  |
